# Towards a computational history of modernism in European literary history: Mapping the Inner Lives of Characters in the European Novel (1840–1920)

**DOI:** 10.12688/openreseurope.16290.1

**Published:** 2023-08-23

**Authors:** Tamara Radak, Lou Burnard, Pieter Francois, Agnes Hilger, Fotis Jannidis, Gábor Palkó, Roxana Patras, Michael Preminger, Diana Santos, Christof Schöch

**Affiliations:** 1 University of Vienna, Vienna, 1010, Austria; 2 Independent researcher, Oxford, England, UK; 3 University of Oxford, Oxford, England, OX1 2JD, UK; 4 University of Würzburg, Würzburg, Bavaria, 97070, Germany; 5 Eötvös Loránd University, Budapest, 1053, Hungary; 6 Alexandru Ioan Cuza University, Iași, 700506, Romania; 7 Oslo Metropolitan University, Oslo, 0130, Norway; 8 University of Oslo, Oslo, 1072, Norway; 9 University of Trier, Trier, Rhineland-Palatinate, 54286, Germany

**Keywords:** distant reading, literary history, European novel, modernism, literary characters

## Abstract

In this paper, we investigate the common narrative in literary history that the inner lives of characters became a central preoccupation of literary modernism. We operationalise this notion via a proxy, tracing the use of verbs relating to inner life across 10 language corpora from the ELTeC collection, which comprises novels from the period between 1840–1920. We expected to find an increase in the use of inner-life verbs corresponding to the traditional periodisation of modernism in each of the languages. However, different experiments conducted with the data do not confirm this hypothesis. We therefore look at the results in a number of more granular ways, but we cannot identify any common trends even when we split the verbs into individual categories, or take canonicity or gender into account. We discuss the obtained results in detail, proposing potential reasons for them and including potential avenues of further research as well as lessons learned.

## Introduction

In 1924, Virginia Woolf famously proclaimed that “on or about December 1910, human character changed” ( 2009: 38) and argued that it was the task and responsibility of modern(ist) fiction to represent the complexities of a character’s interiority through new and innovative literary techniques. Since the introduction of the “inward turn” ( Kahler, 1973), the notion that the “inner life, the soul, *l’âme*, *die Seele*, *sjœlen*” ( Bradbury & McFarlane, 1991: 196) of characters constitutes a central preoccupation of literary modernism has become a critical staple. In line with recent contributions to the understanding of literary characters through distant reading methods (e.g. Freitas & Santos, 2023; Piper, 2023), we aim to test this notion by tracing references to “inner life” in the European Literary Text Collection, a collection created within the COST Action "Distant Reading for European Literary History". ^1^

## Measuring modernism through “inner life”

In the field of Computational Literary Studies, phenomena often cannot be modelled in their entire complexity; therefore, a proxy is considered as indicative of the phenomenon in question. In our case, the hypothesis is based on the abovementioned narrative that the characters’ inner life became central to literary modernism. Hence, we operationalize the use of inner-life verbs, such as *feel* or *think* (see the Methods section), as a proxy. Our assumption has been that these verbs are employed when inner processes are (re)presented and that their use may correspond to the development of literary modernism in each of the languages. The issue at hand seemed a good test case for a comparative computational study of the novel corpora included in ELTeC, ^2^ since the approach allows us to identify and visualise trends appearing at different times and compare similarities and differences across languages. 

We want to emphasize that the terms ‘modernism’, ‘modern’ and ‘modernity’ are not unambiguous, but rather carry fluctuant meanings across national literary histories ( Călinescu, 1987; Friedman, 2001). Some sources locate the start of modernity during Romanticism or even the Renaissance, while others consider only the changes in the literary/aesthetic field around 1890 as ‘modern(ist)’. Additionally, the themes and techniques perceived as ‘modern’ or ‘modernist’ did not develop synchronously throughout Europe. While there is thus no common and/or uncontested periodisation of ‘European Modernism’, we can observe processes implying formal innovation in national literatures throughout Europe at different times. Therefore, we assume that, based on the traditional periodization(s) of modernism in each language, certain upward trends can be observed over time, and we do expect to see an increase at least in those European languages which are commonly considered to be prominent driving forces of ‘modernism’ during the period sampled by ELTeC.

## Data

This study uses 10 ELTeC corpora: English, French, German, Hungarian, Norwegian, Portuguese, Romanian, Serbian, Slovenian, and Spanish. ELTeC was created to reflect the narrative literature (novels) in various languages from 1840 to 1920 in a comparable way. Each of the corpora includes 100 public-domain novels, with diverse metadata (author name, gender, time slots, word count, etc.) that operationalize concepts (e.g. canonicity, reflected in reprint count). Although the editors aimed at balanced collections and fair distribution according to variables, not all collections could comply with the proposed criteria (see Burnard *et al.*, 2021; Herrmann *et al.*, 2020; Schöch *et al.*, 2021). 

For the purpose of the present study, we chose ELTeC – level 2, that is, corpora that were xml texts, TEI encoded and POS-tagged. The size of the full material appears in Table 1. ^3^

**Table 1.** Data overview.

Language	No. works	Reprint Count distribution (frequent/rare)	Word Count	Inner-life verbs
(A) ELTeC				
deu	100	48 / 46	12,738,842	306,040
eng	100	32 / 68	12,227,703	170,431
fra	100	44 / 56	8,712,219	169,719
hun	100	32 / 67	6,948,590	117,661
nor	58	32 / 26	3,686,837	83,794
por	100	26 / 60	6,799,385	115,427
rom	100	24 / 76	5,951,910	230,260
slv	100	48 / 52	5,682,120	112,436
spa	100	46 / 54	8,737,928	137,050
(B) Diachronic corpora				
por_1840-1949	233	n/a	14,882,964	245,334
ger_1760-1920	1147	n/a	114,208,981	933,488
fra_1750-2000	1086	n/a	77,988,445	1,489,292

## Methods

Just as there are many conceptual ways and proxies available to start unpacking the relationship between literary modernism and the use of inner-life verbs, for the methodology, too, we had a range of options to operationalize and then test our hypothesis. The methodology described here has slowly evolved over multiple discussions, during which we carefully weighed advantages and disadvantages for each approach. Our methodology emerged from a trial-and-error process that will have to be repeated/expanded on in larger studies. Even so, the data gathered as part of alternative approaches is available in the supplementary material. Originally, we wanted to compare two approaches: the first based on the methodology described in this paper, with the difference that we simply selected any 10 inner-life verbs rather than 3 verbs from 6 categories. This first attempt was perceived as inferior, as the choices were not underpinned by theoretical literature and differences among languages across categories went unnoticed. Our second approach also had two iterations. The first iteration used seed words (feel, think, believe, know, hope, wish and their translations in the ELTeC languages), augmented by 15 nearest neighbours. This resulted in a list of up to 100 words. However, filtering the noise from this list was, for the moment, beyond the scope of this first paper. The second iteration expanded on this attempt by also using the 6 internal state language categories. ^4^

It is important to see the methodology we settled on in the end, above all, as a first conceptual bloc in a longer debate. Thus, our methodology revolves around two sets of choices: 1) how the items on the language-specific wordlists are selected, and 2) how the data is analysed. The choice of the morphological category is informed by recent literature on “internal state language”, which has been studied intensively, especially in the context of the Theory of Mind framework ( Tompkins *et al.*, 2018). From this framework, we derived six relevant categories:

● **perception:** verbs relating to sensory experience (e.g. “see” something, “listen” to something, “perceive” somebody);
● **physiology:** verbs relating to the body/bodily experience that influences one’s inner life (e.g. “hurt”, “feel hungry”);
● **affect:** verbs relating to emotions or emotional states (e.g. “love”, “hate”)
● **volition and ability:** verbs relating to wishes, desires etc. and/or ability (e.g. “desire”, “wish”) ^5^;
● **cognition:** verbs relating to mental processes (e.g. “remember”, “forget”);
● **moral judgment and obligation**: verbs that contain evaluative statements (e.g. “she preferred x over y”) and/or that refer to an obligation (e.g. “they should be careful”; “he was obliged to her”).

We asked domain experts for each language to go over the entire verb frequency list and select three verbs for each of the abovementioned categories. It is noteworthy that this procedure maximizes diversity within the total of inner-life verbs. If we had simply scored for absolute frequency, the category ‘perception’ would have been dominantly represented. Instead, our approach gives the six categories equal weight and thus also allows us to inspect them individually.

The analysis step is centred around establishing, for each language-based corpus, the prevalence of each inner-life verb. The prevalence in one novel is defined as the proportion of instances of each inner-life verb relative to all instances of verbs used. For this analysis, this prevalence is established (a) for all six categories taken together and (b) for each of the categories separately.

We visualize the data in three ways:

(1) Using a detailed scatterplot, we can show each novel as a function of its publication year and the prevalence of inner-life verbs; this allows for the calculation of a linear regression line that shows whether the prevalence increases or decreases over time.

(2) Using a group of boxplots, each showing the distribution of the prevalence of inner-life verbs during one decade, we summarize the data.

(3) Splitting the data into an earlier (1840–1870) and a later phase (1890–1920), and visualizing the distribution of inner-life verbs as a density plot, we display the degree of overlap and similarity between the data for the earlier and the later period. This also allows for the calculation of a test statistic and the probability that the two distributions are part of the same underlying distribution. Only if this probability is below a threshold (traditionally, p<0.05) should we assume a genuine underlying difference between the distributions of the earlier and later phases in the data. 

## Results

Based on the corpora, lists of verbs and methods of analysis described in the previous sections, we have obtained a set of results in the form of frequency tables and data visualizations. ^6^

**Figure 1.**
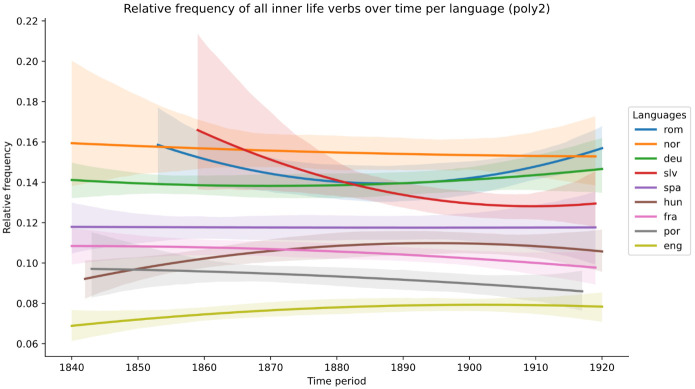
Relative frequency of all inner-life verbs over time, per language (second-order polynomial regression line).

The examination of scatterplots and boxplots for all verbs first shows various slight trends, whether upwards (English, Hungarian), downwards (French, Norwegian, Portuguese and, most markedly, Slovenian) or more or less flat (German, Romanian, Spanish). However, the density plots and tests for statistical significance do not detect any differences with statistical significance. ^7^ For a summary of these data, see Figure 1.

As a consequence, we look at the data in several more fine-grained ways. First, we consider whether individual categories of verbs may display more marked trends than was the case for all verbs taken together. However, this yields similarly inconclusive results for all corpora. Second, we consider whether there is any divergence in the trends according to ELTeC canonicity and gender criteria. ^8^ We only find slight divergences, with strongly overlapping confidence intervals and therefore no statistically significant divergences.

**Figure 2.**
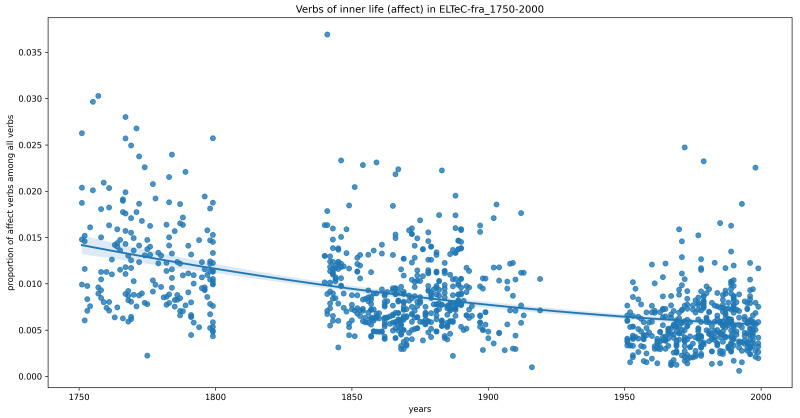
Relative frequency of inner-life verbs (affect category only) in the extended French data (1750–2000), with a second-order polynomial regression line.

Finally, we want to exclude the possibility that we do not see significant trends because the datasets are too small, or because the time period 1840–1920 is too short for such trends to become visible. Therefore, for languages where more data from a wider chronological range is available (French, German, Portuguese), we perform the same analysis. This time, in the French data, we do see statistically-significant downward trends, both overall and most markedly for verbs of affect ( Figure 2). This, however, contradicts our initial expectation of an upward trend.

## Discussion

For the period primarily investigated here (1840–1920), the proportion of inner-life verbs, at least in the way we have defined and operationalized them, appears to be remarkably stable across a number of different languages, corpora, and subsets of the data. Additionally, it appears to be somewhat stable even when considering corpora with a larger diachronic scope. In other words, the data do not confirm our hypothesis about detecting a rising trend, an increase in the use of inner-life verbs. The reason for this finding could be that the assumptions in literary history about this aspect of modernism are false. However, it is probably much too early to reach such a conclusion, because there could be other reasons, for example:

*The time slot could be too short*. As the experiment with the French 1100 novels shows, we can see a trend when we look at the long history from 1750 to 2000, but it is a decreasing one. Conversely, there is no trend visible in either the German or Portuguese data. We assumed that we would find the trend in some languages earlier and in others later, so our time window of 80 years could be too short for the phenomenon we are interested in. However, as the experiments with larger corpora show, this expectation is not supported by the data.

*Canonicity*: The mix of novels in the ELTeC could be misleading. The description of literary history usually refers to a rather small set of texts which represent retrospectively the most advanced trends of their time and which made it into the literary canon. The various corpora also contain non-canonical novels, a category which sometimes refers to texts from high literature (literature meant to be read by educated readers, often employing complex literary devices) that were not considered to be relevant. Sometimes, the category refers to texts which are precursors of popular fiction. But even despite this ambiguity, the meaning of canonicity is rather clear, and if we consider only these novels, we can see that in the French and Portuguese data, the trend decreases, while there is no trend for the German corpus and only small trends for the Spanish one. Only in the English data we see exactly the upwards trend we expected.

*Confounding variables*: The distribution of narrative devices is certainly not stable for the time period we are interested in, as writers developed innovative techniques that challenged “ill-fitting” ( Woolf, 2009) realist forms of literature. There are two connected trends described by literary history: the disappearance of the narrator (at least a trend to avoid third-person narrators or strong commenting voices) and the trend to prefer showing and avoid telling ( Klauk & Köppe, 2014). The latter trend has not only been confirmed but shown to be visible in the history of the novel between 1750 and 1950 ( Heuser & Le-Khac, 2012). The French long-term data show a decrease of verbs indicating a description of affects, which seems to conform to this tendency. Conversely, although we know from other experiments that the German novel is also changing in the direction of a preference for more concrete expressions, we cannot see any long-term trend with respect to the inner-life verbs.

While acknowledging recent work that challenges the notion of the “inward turn” in modernist literature ( Conroy, 2014; Gang, 2013; Herman, 2011; Miguel-Alfonso, 2020), there are good reasons to conduct further research regarding the assumption that modernism comes with an increased interest in the inner life of characters. At the same time, it has become very clear that the European history of the novel is not just a trickling down of modernism from the centre to the periphery: we see very different trends in the data, even if we grant something like a time lag between national developments. This indicates that modern interests and sensibilities were integrated into very different national histories and any attempt to tell this story on the European level must start from a more complex model of literary history.

## Conclusion: Lessons learned and further research

By using corpora in 10 different European languages, we have attempted to operationalize the hypothesis that characters’ inner lives become a central concern of literary modernism.

This has been an exploratory endeavour, partly owing to the multilingual feature of the ELTeC. Several factors may have been responsible for the inconclusiveness of our results. Some might be related to the materials worked with, as discussed in the results and discussion sections:

- the size and scope of the collection
- the fact that ‘modern’, ‘modernist’, ‘modernism’, as well as associated questions of periodisation, differ across languages and national literatures

However, the inconclusiveness may also owe to methodological choices:

- Were we sufficiently ‘deep’ in our approach? Provided the verbs are indeed indicative of inner life in all our languages: is the occurrence pattern of these verbs more complex than a pure counting of relative occurrences or even than ‘the company they keep’ (traceable through their embedding-neighborhood) ^9^?
- Have we been paying enough attention to the different distributions of the categories in the different languages?

With regard to these points, a comparative study employing similarly complex tools and methods as Piper, 2023 (bookNLP, “super-sense tags”) might provide a further potentially fruitful avenue of research. With its focus on embodiment, rather than emotional states, in the Hathi1M corpus, Piper’s paper proceeds from a diametrically opposed starting point, yet arrives at a similar result that corroborates our findings: While verbs referring to “embodiment”, in particular those referring to “motion”, experienced a steady rise in the period between 1800 and 2000, verbs of cognition do not display a similar upward trend ( Piper, 2023: 7).

Finally, working together in a multilingual and interdisciplinary setting is, no doubt, a very rewarding and enriching endeavour. At the same time, it entails its own challenges regarding explicit or implicit expectations, conventions, and terminologies that are important to consider when embarking on comparable projects. 

## Data Availability

Data, code and results relevant to this paper are made openly available at https://github.com/COST-ELTeC/innerlife/ (DOI: https://doi.org/10.5281/zenodo.8189812). All raw data in the repository is made available with a Creative Commons Zero (CC0) licence. All figures and code in the repository are made available with a Creative Commons Attribution 4.0 International CC BY licence.
