# Towards a computational history of modernism in European literary history: Mapping the Inner Lives of Characters in the European Novel (1840–1920)

**DOI:** 10.12688/openreseurope.16290.2

**Published:** 2024-03-20

**Authors:** Tamara Radak, Lou Burnard, Pieter Francois, Agnes Hilger, Fotis Jannidis, Gábor Palkó, Roxana Patras, Michael Preminger, Diana Santos, Christof Schöch

**Affiliations:** 1 University of Vienna, Vienna, 1010, Austria; 2 Independent researcher, Oxford, England, UK; 3 University of Oxford, Oxford, England, OX1 2JD, UK; 4 University of Würzburg, Würzburg, Bavaria, 97070, Germany; 5 Eötvös Loránd University, Budapest, 1053, Hungary; 6 Alexandru Ioan Cuza University, Iași, 700506, Romania; 7 Oslo Metropolitan University, Oslo, 0130, Norway; 8 University of Oslo, Oslo, 1072, Norway; 9 University of Trier, Trier, Rhineland-Palatinate, 54286, Germany

**Keywords:** distant reading, literary history, European novel, modernism, literary characters

## Abstract

In this paper, we investigate the common narrative in literary history that the inner lives of characters became a central preoccupation of literary modernism – a phenomenon commonly referenced as the “inward turn”. We operationalize this notion via a proxy, tracing the use of verbs relating to inner life across 10 language corpora from the ELTeC collection, which comprises novels from the period between 1840–1920. We expected to find an increase in the use of inner-life verbs corresponding to the traditional periodisation of modernism in each of the languages. However, different experiments conducted with the data do not confirm this hypothesis. We therefore look at the results in a number of more granular ways, but we cannot identify any common trends even when we split the verbs into individual categories, or take canonicity or gender into account. We discuss the obtained results in detail, proposing potential reasons for them and including potential avenues of further research as well as lessons learned.

## Introduction

In 1924, Virginia Woolf famously proclaimed that “on or about December 1910, human character changed” ( 2009: 38), arguing that it was the task and responsibility of modern(ist) fiction to represent the complexities of a character’s interiority through new and innovative literary techniques. Since the introduction of the “inward turn” ( Kahler, 1973), the notion that the “inner life, the soul, *l’âme*, *die Seele*, *sjœlen*” ( Bradbury & McFarlane, 1991: 196) of characters constitutes a central preoccupation of literary modernism has become a critical staple. In line with recent contributions to a better understanding of literary characters through distant reading methods (e.g. Freitas & Santos, 2023; Piper, 2023), we aim to test this notion by tracing references to verbs denoting “inner life” in the European Literary Text Collection (ELTeC), a multilingual collection of corpora created within the COST Action "Distant Reading for European Literary History". ^1^ Modelling complex, occasionally even contradictory developments and processes in the European novel is beyond the scope of a single paper and a number of confounding variables need to be considered. The primary goal of this brief report is to illuminate the choices that went into the project, reflect on potential confounding variables and outline challenges encountered, for the benefit of future researchers aiming to work with ELTeC and/or explore distant reading methods in the context of literary periodisation.

## Modernism and the “inward turn”

In a 2014 article, Melanie Conroy notes that “[t]he alleged inward turn of the modernist novel has become one of the great truisms of academic literary criticism” ( 2014: 121). Conroy employs a combination of distant and close reading methods to critically examine this commonplace, tracing the use of “mental verbs” ( 2014: 117) across French literary texts between 1800 and 1929 and asking, “do reporting clauses and mental verbs occur more frequently in some authors, texts, or decades than elsewhere? If the frequency of these markers is significantly higher in them, these authors, books, or decades quite possibly engage in more thought representation and thereby strengthen the ‘inward turn’” ( Conroy, 2014: 134). The present paper builds and expands on Conroy’s central points and findings in two ways: firstly, it addresses the call to investigate representations of mental states in different languages and literatures ( Conroy, 2014: 125) by working with the multilingual text collection ELTeC; secondly, we similarly focus on mental verbs, but develop the approach further by drawing on a six-part categorisation system derived from the “Theory of Mind” framework ( Bretherton & Beeghly (1982); Tompkins *et al*. (2019)). Thereby, we diversify the mental verbs chosen beyond the two that Conroy primarily focusses on, “he/she thought” and “said to themselves”. ^2^ One of our aims is furthermore to examine how Conroy’s findings that a “significant increase in the use of mental verbs” can be observed, but that “the two pointers of represented thought at issue *did not increase in a linear fashion* in the period 1800–1929” ( Conroy, 2014: 140; emphasis added) in the French corpus compares to results from a multilingual collection such as ELTeC.

Taking the common critical narrative that the characters’ inner lives became central to literary modernism as a starting hypothesis to be critically investigated, in the present paper, we operationalize the use of inner-life verbs, such as *feel* or *think* (see the Methods section) in ELTeC as a proxy. Our assumption is that these verbs are employed when interior processes are represented and that upwards or downwards trends in their use may roughly correspond to developments in European literary history. The issue at hand seemed cut out for a comparative computational study of the novel corpora included in ELTeC, ^3^ since this approach allows us to identify and visualise trends appearing at different times and compare similarities and differences across languages. 

We want to emphasize that the terms ‘modernism’, ‘modern’ and ‘modernity’ are not unambiguous, but rather carry fluctuant meanings across national literary histories ( Călinescu, 1987; Friedman, 2001). Some sources locate the start of modernity during Romanticism or even the Renaissance, while others consider only the changes in the literary/aesthetic field around 1890 as ‘modern(ist)’. Additionally, the themes and techniques perceived as ‘modern’ or ‘modernist’ did not develop synchronously throughout Europe. While there is thus no common and/or uncontested periodisation of ‘European Modernism’, we can observe processes involving formal innovation in national literatures throughout Europe at different times. Based on commonly accepted (though not entirely undisputed) periodisations of modernism in each of the national literatures represented, we would expect to see, for example, an upward trend in mental verbs between the late 19th and the early to mid-20th century in the English-, Serbian- and Romanian-speaking corpora, and the late 19th century in the German- and Norwegian-speaking corpora. The Portuguese corpus from the ELTeC collection is included as a test case for the above-mentioned hypothesis: While Portuguese modernism is said to have started with the *Revista Orpheu* (in 1915), no authors that would be considered hallmark modernist writers (for instance, Almada Negreiros, António Ferro) could be included in the collection for copyright reasons. Therefore, we expect the results from the Portuguese corpus to differ from those of the other corpora and include it as a counter example.

## Data

This study uses 10 ELTeC corpora: English, French, German, Hungarian, Norwegian, Portuguese, Romanian, Serbian, Slovenian, and Spanish. ELTeC was created to reflect a sample of novels in various languages (the core collection consisting of 10, the expanded collection of 20 languages) from 1840 to 1920 based on criteria that ensured “rough comparability” ( Schöch *et al.*, 2021: 4) across subcollections. Each of the corpora includes 100 public-domain novels, with diverse metadata (author name, gender, publication date, word count, etc.) that operationalize certain concepts (e.g. canonicity, reflected in reprint count). Although the editors aimed at balanced subcollections and fair distribution according to variables, not all corpora could comply with the proposed criteria (for detailed discussions of the selection criteria and the process of corpus-building, see Burnard *et al.*, 2021; Herrmann *et al.*, 2020; Schöch *et al.*, 2021). 

For the purpose of the present study, we chose ELTeC – level 2, that is, corpora that contain XML texts, which are TEI encoded and POS-tagged. The size of the full material appears in Table 1. ^4^

**Table 1.** Data overview.

Language	No. works	Reprint Count distribution (frequent/rare)	Word Count	% of inner-life verbs per words	Inner-life verbs (absolute frequency)
(A) ELTeC					
Deu	100	48 / 46	12,738,842	2.4	306,040
Eng	100	32 / 68	12,227,703	1.39	170,431
Fra	100	44 / 56	8,712,219	1.95	169,719
Hun	100	32 / 67	6,948,590	1.69	117,661
Nor	58	32 / 26	3,686,837	2.27	83,794
Por	100	26 / 60	6,799,385	1.7	115,427
Rom	100	24 / 76	5,951,910	3.87	230,260
Slv	100	48 / 52	5,682,120	1.98	112,436
Spa	100	46 / 54	8,737,928	1.57	137,050
(B) Diachronic corpora					
por_1840-1949	233	n/a	14,882,964	1.65	245,334
ger_1760-1920	1147	n/a	114,208,981	0.82	933,488
fra_1750-2000	1086	n/a	77,988,445	1.9	1,489,292

## Methods

Just as there are many conceptual ways and proxies available to start unpacking the complex relationship between literary modernism, the inward turn and the use of inner-life verbs, we had a range of options to explore and discuss when operationalizing and then testing our hypothesis. The methodology described here has slowly evolved over multiple discussions, during which we carefully weighed advantages and disadvantages for each approach, together with domain experts for each language, i.e. collaborators from the COST Action who had expertise in a given national literature and/or were native speakers of the respective language. Our methodology emerged from a trial-and-error process that will have to be repeated and expanded on in larger studies. Even so, the data gathered as part of alternative approaches is available in the supplementary material. Originally, we wanted to compare two approaches: the first based on the methodology described in this paper, with the difference that we simply selected the 10 most frequent inner-life verbs in the respective corpus rather than 3 verbs from 6 categories. Our second – eventually abandoned – approach used seed words (feel, think, believe, know, hope, wish and their translations in the ELTeC languages), augmented by 15 nearest neighbours found through word embeddings. This resulted in a list of up to 100 words. However, filtering the noise from this list was, for the moment, beyond the scope of this first paper. ^5^ We eventually decided to return to the first approach, but provide a clearer theoretical basis for the selection of verbs by using existing categories from theoretical literature on inner-life language.

Our methodology revolves around two sets of choices: 1) how the items on the language-specific wordlists are selected, and 2) how the data is analysed. It is presented as a first conceptual bloc in a much longer, complex debate. The choice to focus on the morphological category of verbs is based on our aim to compare the results obtained from a multilingual collection to Conroy (2014)’s findings from a single-language corpus. The selection of these verbs is informed by recent literature in psychology on “internal state language”, which has been studied intensively, especially in the context of the Theory of Mind framework ( Bretherton & Beeghly, 1982; Tompkins *et al.*, 2019). Drawing on Bretherton and Beeghly’s grouping of utterances relating to mental states, we derived six relevant categories that were used when compiling the list of verbs to be mapped:

● **perception:** verbs relating to sensory experience (e.g. “see” something, “listen” to something, “perceive” somebody);
● **physiology:** verbs relating to the body/bodily experience that influences one’s inner life (e.g. “hurt”, “feel hungry”);
● **affect:** verbs relating to emotions or emotional states (e.g. “love”, “hate”)
● **volition and ability:** verbs relating to wishes, desires etc. and/or ability (e.g. “desire”, “wish”) ^6^;
● **cognition:** verbs relating to mental processes (e.g. “remember”, “forget”);
● **moral judgment and obligation**: verbs that contain evaluative statements (e.g. “she preferred x over y”) and/or that refer to an obligation (e.g. “they should be careful”; “he was obliged to her”).

We asked domain experts for each language to go over the entire verb frequency list and select the three most frequent verbs for each of the abovementioned categories. Primarily due to the complexities of syntactic parsing for all these languages, filtering the verbs through dependency parses or similar could not be done reliably across languages. We normalized counts of inner-life verbs against the overall verb count for best possible comparability. This procedure maximizes diversity within the total of inner-life verbs, ensuring that the selected verbs have a high overall relative frequency in a given language corpus. If we had used pre-established seed words for this method, the results might have been skewed because specific verbs may be used more frequently in some languages than in others. Through this broader approach, based on scientifically derived categories, we believe that we have arrived at comparable – though of course not identical – lists that better represent the idiosyncracies of the respective corpora (and associated languages). If we had simply scored for absolute frequency, the category ‘perception’ would have been dominantly represented. Instead, our approach gives the six categories equal weight and thus also allows us to inspect them individually.

The analysis consists of establishing the prevalence of each inner-life verb from the list for each language-based corpus. The prevalence in one novel is defined as the proportion of instances of each inner-life verb relative to all instances of verbs used. All analyses are performed (a) for all six categories taken together and (b) for each of the categories separately.

We visualize the data in three ways:

(1) Using a detailed scatterplot, we can show each novel as a function of its publication year and the prevalence of inner-life verbs; this allows for the calculation of a second-order polynomial regression line that shows whether the prevalence increases or decreases over time.

(2) Using a group of boxplots, each showing the distribution of the prevalence of inner-life verbs during one decade, we summarize the data.

(3) Splitting the data into an earlier (1840–1870) and a later phase (1890–1920), and visualizing the distribution of inner-life verbs as a density plot, we display the degree of overlap and similarity between the data for the earlier and the later period. This also allows for the calculation of a test statistic and the probability that the two distributions are part of the same underlying distribution. Only if this probability is below a threshold (traditionally, p<0.05) should we assume a genuine underlying difference between the distributions of the earlier and later phases in the data. 

## Results

Based on the corpora, lists of verbs and methods of analysis described in the previous sections, we have obtained a set of results in the form of frequency tables and data visualizations. ^7^

**Figure 1.**
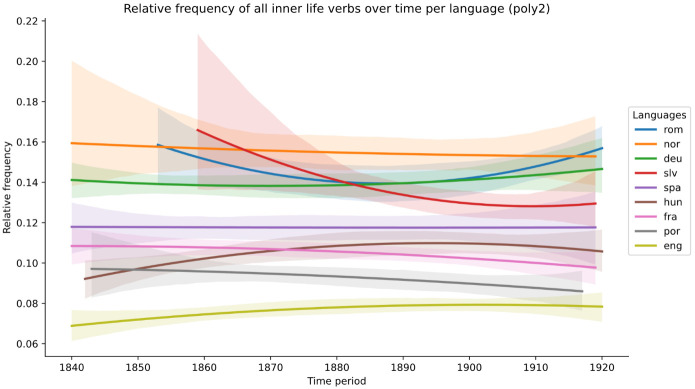
Relative frequency of all inner-life verbs over time, per language (second-order polynomial regression line).

The examination of scatterplots and boxplots for all verbs first shows various slight trends, whether upwards (English, Hungarian), downwards (French, Norwegian, Portuguese and, most markedly, Slovenian) or more or less flat (German, Romanian, Spanish). However, the density plots and tests for statistical significance do not detect any differences of statistical significance. ^8^ For a summary of these data, see Figure 1.

As a consequence, we look at the data in several more fine-grained ways. First, we consider whether individual categories of verbs may display more marked trends than was the case for all verbs taken together. However, this yields similarly inconclusive results for all corpora. Second, we consider whether there is any divergence in the trends according to ELTeC canonicity and gender criteria. ^9^ We only find slight variations between them, with strongly overlapping confidence intervals and therefore no statistically significant divergences.

**Figure 2.**
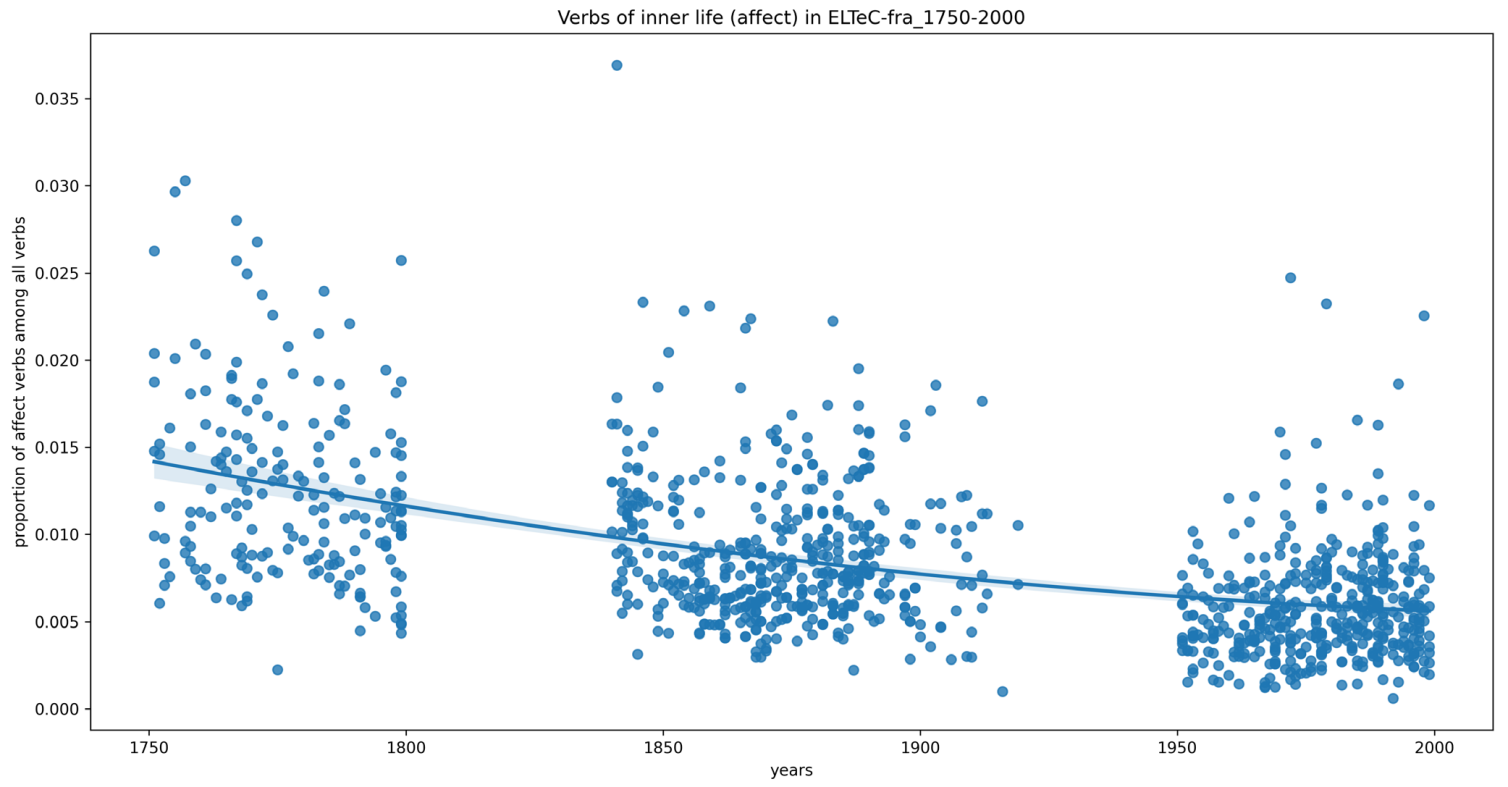
Relative frequency of inner-life verbs (affect category only) in the extended French data (1750–2000), with a second-order polynomial regression line.

Finally, we want to exclude the possibility that we do not see significant trends because the datasets are too small, or because the time period 1840–1920 is too short for such trends to become visible. Therefore, for languages where more data from a wider chronological range is available (French, German, Portuguese), we perform the same analysis. This time, in the French data, we do see statistically-significant downward trends, both overall and most markedly for verbs of affect ( Figure 2) ^10^. This result, however, contradicts our initial expectation of an upward trend.

## Discussion

For the period primarily investigated here (1840–1920), the proportion of inner-life verbs, in the way we have defined and operationalized them, appears to be remarkably stable across a number of different languages, corpora, and subsets of the data. Additionally, their distribution appears to be statistically stable in all but one language even when considering corpora with a larger diachronic scope. In other words, the data do not confirm the original hypothesis of a rising trend in the use of inner-life verbs over time. The reason for this finding could be that assumptions in literary history about this aspect of modernism are false. However, it is probably much too early to reach such a conclusion, because there could be other reasons, for example:

*The time slot represented in ELTeC could be too short*. As the experiment with the 1100 French novels shows, we can see a trend when we look at the long history from 1750 to 2000, but it is a decreasing one. Conversely, there is no trend visible in either the German or Portuguese temporally extended corpus. We assumed that we would find an upward trend earlier in some languages and later in others, so our time window of 80 years could be too short for the phenomenon we are interested in. However, as the experiments with larger corpora show, this expectation is not supported by the data.

*Canonicity*: The mix of novels in ELTeC could be misleading. Traditional accounts of literary history are usually based on a rather small set of texts which represent retrospectively the most advanced trends of their time and which made it into the literary canon. The various ELTeC corpora also contain non-canonical novels, a category which sometimes refers to texts from high literature (literature meant to be read by educated readers, often employing complex literary devices) that were excluded from canonical accounts of a given national literature for various reasons. Sometimes, the category refers to texts which are precursors of popular fiction. But even despite this ambiguity, the meaning of canonicity is operationalized according to specific parameters in ELTeC. If we consider only canonical novels from the selected corpora, we can see a decreasing trend in the French and Portuguese data, while there is no trend for the German corpus and only small trends for the Spanish one. ^11^ Only in the English data do we see exactly the upwards trend we expected. ^12^

*Confounding variables*: The distribution of narrative devices is certainly not stable for the time period we are interested in, as writers developed innovative techniques that challenged “ill-fitting” ( Woolf, 2009) realist forms of literature and sought new ways of capturing the ever-elusive complexity of human character. Two connected trends are often mentioned in literary history: the disappearance of the narrator (at least a trend to avoid third-person narrators or strong commenting voices) and a preference for showing as opposed to telling ( Klauk & Köppe, 2014). The latter trend has not only been confirmed but shown to be visible in the history of the novel between 1750 and 1950 ( Underwood, 2019; see also Heuser & Le-Khac, 2012). The French long-term data show a decrease of verbs indicating a description of affects, which seems to conform to this tendency. Conversely, we cannot see any long-term trend with respect to the inner-life verbs in the expanded German corpus. Interestingly, Conroy’s French corpus does not seem to confirm the aforementioned trend towards a preference for showing rather than telling, as she notes that “common reporting clauses and mental verbs appeared in a wide variety of texts, [...] alongside other, 'free' techniques” ( 2014: 117). This parallel development could potentially be considered a confounding variable insofar as the use of both techniques in equal or similar measure might mean that neither appears as a statistically significant trend in the data.

In its critical reconsideration of the common narrative of the “inward turn” as a key aspect of modernist literature, Conroy’s article can be seen in the context of other recent publications ( Gang, 2013; Herman, 2011; Miguel-Alfonso, 2020) which similarly highlight the complexity of issues surrounding periodisation based on formal, aesthetic, and thematic features and the need for a nuanced engagement with them. Both the insights from these recent approaches and the lack of an unequivocal trend in our data suggest that there are good reasons to conduct further research into the manifold connections between literary modernism and the inner life of characters, which eschew simple and univocal narratives. At the same time, our data show that the European history of the novel is not just a trickling down of modernism from the centre to the periphery, as it has traditionally often been conceived (a view that is increasingly being challenged in recent conceptualisations, of which Friedman’s *Planetary Modernisms* (2015) is a key representative). We see very different trends in the data, even if we grant a potential time lag between national developments. This indicates that modern interests and sensibilities were integrated into very different national histories and any attempt to tell this story on the European level must start from a more complex model of literary history.

## Conclusion: Lessons learned and further research

By tracing the use of inner-life (or mental) verbs, based on 6 categories derived from the “Theory of Mind” framework, across ELTeC corpora in 10 different European languages, we have attempted to operationalize the hypothesis that characters’ inner lives become a central concern of literary modernism. The methodology chosen in this paper has expanded on the approach adopted by Conroy (2014) by investigating the “inward turn” in a multilingual collection and by further specifying and diversifying the categories according to which the mental verbs were selected. Largely in accordance with Conroy’s findings, but contrary to expectations based on the common critical narrative of the “inward turn” as an essential feature of literary modernism, our data do not show an increase in the use of these verbs over time, but rather a relatively stable distribution of them within the period in question.

This project has been an exploratory endeavour, partly owing to the multilingual feature of ELTeC and the lack of existing cross-linguistic frameworks and methods that might fully encapsulate the complex tension between different national and cross-national developments in European literary history. Several factors may have been responsible for the inconclusiveness of our results. Some might be related to the materials worked with, as discussed in the results and discussion sections:

- the size and scope of the collection
- the fact that ‘modern’, ‘modernist’, ‘modernism’, as well as associated questions of periodisation, differ across languages and national literatures

However, the inconclusiveness may also owe to methodological choices:

- Were we sufficiently ‘deep’ in our approach? Provided the verbs are indeed indicative of inner life in all our languages, is the occurrence pattern of these verbs more complex than a pure counting of relative occurrences? One avenue of future research would be applying word embedding-analysis to the texts and trying to tease out the contexts that the seed words occur within or even ‘the company they keep’ (traceable through their embedding-neighborhood). ^13^
- Have we been paying enough attention to the different distributions of the categories in the different languages?

With regard to these points, a comparative study employing similarly complex tools and methods as Piper, 2023 (bookNLP, super-sense tagging) might provide a further potentially fruitful avenue of research. With its focus on embodiment, rather than emotional states, in the Hathi1M corpus, Piper’s paper proceeds from a diametrically opposed starting point, yet arrives at a similar result that corroborates our findings: While verbs referring to “embodiment”, in particular those referring to “motion”, experienced a steady rise in the period between 1800 and 2000, verbs of cognition do not display a similar upward trend ( Piper, 2023: 7).

Finally, working together in a multilingual and interdisciplinary setting is, no doubt, a very rewarding and enriching endeavour. At the same time, it entails its own challenges regarding explicit or implicit expectations, conventions, and terminologies that are important to consider when embarking on comparable projects. 

## Data Availability

Data, code and results relevant to this paper are made openly available at https://github.com/COST-ELTeC/innerlife/ (DOI: https://doi.org/10.5281/zenodo.8189812). All raw data in the repository is made available with a Creative Commons Zero (CC0) licence. All figures and code in the repository are made available with a Creative Commons Attribution 4.0 International CC BY licence.
