# Supplementary material for: Towards a computational history of modernism in European literary history: Mapping the Inner Lives of Characters in the European Novel (1840–1920)
Source: Open Res Eur. 2024 Mar 20;3:128. Originally published 2023 Aug 23. [Version 2] doi: 10.12688/openreseurope.16290.2 (PMC10682596; doi:10.12688/openreseurope.16290.2)
Supplement: Peer review history

# Reviewer report: Reviewer response for version 2

**DOI:** 10.21956/openreseurope.18860.r39037

**Published:** 2024-05-09

**Authors:** Andreas van Cranenburgh, Noa Visser Solissa

**Affiliations:** 1 Assistant professor of Digital Humanities and Information Sciences, University of Groningen, Groningen, Netherlands Antilles; 2 University of Groningen, Groningen, Groningen, The Netherlands

We are pleased to see that our comments concerning the selection of verbs, the word embeddings and the statistical methodology have been addressed and clarified.  We are satisfied with the changes that have been made. 

 We understand the authors’ reasoning that the LIWC lexicon is not suitable for their set up, and their concern about whether LIWC has been validated on comparable corpora. 

 Lastly, we are glad to see that the possible explanation for the results, i.e., that modernism gives more attention to inner life not through explicit inner life verbs but through devices such as free indirect discourse, is discussed in more detail in the revised version.

Is the work clearly and accurately presented and does it cite the current literature?

Partly

If applicable, is the statistical analysis and its interpretation appropriate?

Yes

Are the conclusions drawn adequately supported by the results?

Yes

Are all the source data underlying the results available to ensure full reproducibility?

Yes

Is the study design appropriate and is the work technically sound?

Yes

Reviewer Expertise:

Computational linguistics, computational literary studies.

We confirm that we have read this submission and believe that we have an appropriate level of expertise to confirm that it is of an acceptable scientific standard.

# Reviewer report: Reviewer response for version 1

**DOI:** 10.21956/openreseurope.17586.r35113

**Published:** 2023-11-27

**Authors:** David Mimno, Rebecca Hicke

**Affiliations:** 1 Cornell University, Ithaca, New York, USA; 2 Computer Science, Cornell University, Ithaca, New York, USA

Major Points: 

 The article studies whether the theorized increasing focus on the ‘inner-lives’ of characters associated with modernism can be quantitatively shown by measuring the relative prominence of ‘inner-life’ verbs over time. The authors study this phenomenon in ELTeC corpora for 10 languages. After identifying three ‘inner-life’ verbs from each of six relevant categories for each of the ten languages, they measure the usage of these verbs as a proportion of all verbs each year from 1840-1920. The proportion of ‘inner-life’ verbs used over time does not change significantly over the time period, except for a larger corpus of French literature, in which they find a decrease in the usage of ‘inner-life’ verbs.

 Given that Open Research Europe seems to be a broad repository venue rather than a specific journal, I am uncertain of the expected level of contribution or intended audience for this piece. My comments assume an audience of Digital Humanities researchers and those interested in DH work. Overall, the work seems sound if not particularly exciting. I can see it as a baseline for a more sophisticated study, but at present I feel that the work would be too preliminary to publish in a competitive journal or conference.

 My main criticism is that the assumption that inner life can be characterized by specific verbs, and more importantly that those verbs indicate descriptions of inner life, seems thin and reductive. Does Catullus not hate and love? At this point we have language models that can operate in multiple European languages and extract high-level conceptual constructs. Counting verbs seems somehow both unambitious and unworthy of the subtlety of the concept of inner life. If this were presented as a relatively weak opening play at operationalizing inner life to be empirically disproved, and to be followed by speculation about stronger methods, I would feel more confident. At least looking at objects and subjects in addition to verbs might provide more variation.

 An alternative, and perhaps stronger motivation might be to clarify that even if an observation does not define a construct, it can still serve as an indicator of the presence of the construct. The famous Stanford lit lab result that the Gothic novel is characterized by greater use of locative prepositions does not mean that those prepositions are what makes the novel Gothic, it is that the Gothic is a distinct and noticeable phenomenon that has innumerable statistical manifestations, of which prepositions are just the most countable. "Inner life" verbs could be similar, and they are not.

 Another potential threat to validity is the composition of the corpus. I believe that this collection was developed for general purposes and not for this specific study. I don't know anything about the novels except their rough publication date and the fact that they were prominent enough to be digitized and selected for these collections. Do they represent modernism? Could the early ones have been chosen because they are avant garde (and thus happen to have more modern features), or the later ones because they are in a more conservative prestige-literary tradition? I don't expect so, but the point is that I don't know.

 There are well-known large-scale patterns in literature (at least in English) over this time period, see Ted Underwood's work on narrative time measurements. Over the 19th century English-language literature shifts from a more narrative style to a more dialog-oriented style. I wonder if there are 

 I am also concerned that the choice of only three verbs from each ‘inner-life’ category could skew results. If certain authors favor particular verbs over others or there was a transition from using one verb to another over time – both easily imaginable scenarios – the given results would not reflect the phenomenon being studied. It is particularly concerning to me that the criteria for choosing these verbs is not given. To account for the concern that studying all verbs would skew the results in favor of one category of verb, one could report general trends for all verbs and then individual trends for each category, thus allowing for both a broad and fine-grained study of the phenomenon.

 I don't know how the confidence regions in Figure 1 are defined, but they look like bootstrap CIs, which would be a good option to increase statistical reliability. The second-order curve fit may be too smooth, I would rather see this as the actual numbers in a line plot with per-decade confidence bars.

 Finally, and less importantly, I found the paper difficult to follow in places and believe it could benefit from some reorganization and simplification of language. I comment more on this in the section below. 

 Introduction 

 A final word describing what COST Action “Distant Reading for European Literary History” is (project?) would help clarify for those unfamiliar. 

 Measuring modernism through “inner life” 

 This section feels slightly disjointed; I think that splitting its content between the Introduction and Methods sections would help. Specifically, moving the hypothesis and discussion of modernism to the Introduction and the portion on methods to the Methods section would likely increase flow. 

 The second sentence of the section mentions a hypothesis before one is given which feels odd although the hypothesis can be guessed. 

 The next sentence seems to tease the methods without providing much information on them – this could just be moved to Methods. 

 In the sentence beginning “Our assumption…”, “has been” should be changed to “is” or “was” for clarity.

 The phrase “seemed a good test case” feels overly informal and implies this project was just created to use ELTeC. Perhaps rephrase to say that an additional benefit of the experiment was that it was an opportunity to explore the uses of ELTeC. 

 Adding some clarification to what the “upward trends” referenced in the final paragraph of this section are would be helpful! I assume this means upwards trends in the usage of inner-life verbs, but it would help to have that explicitly stated. 

 Data 

 The sentence beginning “ELTeC was created…” reads slightly awkwardly and may benefit from rephrasing. Specifically, including a specific number instead of saying “in various languages” would be beneficial. The phrase “in a comparable way” sits awkwardly and could be removed. 

 Writing “ELTeC level 2 [corpora or texts]”  instead of “ELTeC  – level 2” may help those unfamiliar with the corpora. 

 “… corpora that were xml texts” should be “corpora containing XML texts, which are TEI encoded and…” 

 Why are the results for Serbian, referenced in the footnote, not included if they show similar trends? 

 Methods 

 The first sentence of the sentence is confusing and may benefit from reorganization. Specifically, “unpacking the relationship between literary modernism and the use of inner-life words” is referenced but I am not sure where this has been discussed in the paper so far. 

 The discussion of how you settled on the methodology (beginning with the sentence “The methodology described here…” and ending with “The second iteration…”) could be significantly shortened or cut. The way the general process described – iterative refinement through testing and discussion – seems standard of this kind of research project. Instead, the section could be formatted as “we chose these methods instead of --- because of ----” or the paper cited in footnote 4 could just be referenced. 

 The sentence beginning “It is important to see the methodology…” distracts from the intention of this paragraph and could be cut. Beginning with “Our methodology revolves around…” would be more impactful. 

 I’m not sure what “the morphological category” is at the point it is mentioned. 

 The criteria by which the language experts selected the verbs for analysis is important. 

 The sentence which starts “The analysis step is centred around…” is confusing and can be cut. 

 It feels odd to have the different visualizations listed before they are shown, particularly because not all of the visualizations are used in the upcoming analysis. Perhaps just mention visualizations in the results when they are used. For the description of the third visualization category, what test statistic is calculated? A technical audience will also know what significance is and the traditional thresholds for p-values; including it is unnecessary. 

 Table 1 and Figure 1 

 I would change the language codes to full language names to increase legibility. 

 Results 

 The first paragraph can be cut. 

 In reference to Footnote 6: I would include any visualizations referenced in the paper. Or, if there is only space for Figure 1, it seems it can be referred to for all of your analysis in this section. 

 “…any differences with statistical significance…” should be “…any differences of statistical significance…” 

 The paragraph beginning “as a consequence” could likely be condensed to one sentence per tested theory. 

 Discussion 

 In the first sentence, the phrase “at least in the way we have defined and operationalized them” undercuts your analysis and its contents are clear from the framing. 

 The word “somewhat” in sentence two feels vague; perhaps you could say “statistically stable in all but one language”? 

 The sentences which begin “The reason for this finding…” and “However, it is probably…” contain a lot of hedging and could likely be combined.  

 The time slot could be too short 

 Moving the sentence about the German and Portuguese data to the end would  improve flow. 

 Canonicity 

 “in the ELTeC” should be “in the ELTeC corpora” 

 I’m not sure what is meant by “The description of literary history.” Common  discussions of literary history?

 The distinction between canonical novels and those from high literature is not  entirely clear to me. Who considers these books irrelevant? 

  “…the meaning of canonicity is clear…” does not hold true in my experience.  

 When stating “we only consider these novels” do you mean only the canonical  novels? If so, how are those determined? 

 Confounding variables 

 Are the “two connected trends” those that could explain the results? Some  qualifier about what these trends are / how they were chosen would be helpful. 

 Please cite or include the “other experiments” referenced. It would be particularly  valuable to know what method for measuring inner-life verbs are used in these experiments. 

 The sentence beginning “While acknowledging…” is confusing and could benefit from rephrasing. 

 What is the “centre” and what is the “periphery”? 

 Instead of “we see,” I would write “our results show” so that it is clear what leads to this conclusion. 

 I am not sure how the data show that “modern interests and sensibilities were integrated into very different national histories. I fully believe this is true, but struggle to follow how the data demonstrate this. 

 Conclusion 

 Why does the multilingual. Nature of ELTeC make this an exploratory endeavor? 

 Saying “provided the studied verbs” or similar instead of “provided the verbs” would add clarity. 

 “… such Piper 2023” should be “such as Piper 2023” 

 The final paragraph commenting on interdisciplinary research may be obvious to an audience used to such collaborations.

Is the work clearly and accurately presented and does it cite the current literature?

Yes

If applicable, is the statistical analysis and its interpretation appropriate?

Yes

Are the conclusions drawn adequately supported by the results?

Yes

Are all the source data underlying the results available to ensure full reproducibility?

Yes

Is the study design appropriate and is the work technically sound?

Yes

Reviewer Expertise:

Digital humanities, text mining

We confirm that we have read this submission and believe that we have an appropriate level of expertise to confirm that it is of an acceptable scientific standard.

# Response

**Published:** 2024-03-05

**Authors:** Tamara Radak

**Affiliations:** English and American Studies, University of Vienna, Vienna, Vienna, Austria

We thank the reviewers for the perceptive and fine-grained report. We appreciate the productive feedback and clearly outlined questions, suggestions, and recommendations and respond to individual points made in the report, outlining how they are addressed in the revised version of the paper below.

- *“ **My main criticism is that the assumption that inner life can be characterized by specific verbs, and more importantly that those verbs indicate descriptions of inner life, seems thin and reductive.**”*

In the revised version, we expanded the section concerning methodology and aimed to clarify our approach further, attempting to answer the important questions raised in the report in the process. We expanded on the connection between our project and Melanie Conroy’s 2014 paper “Before the ‘Inward Turn’: Tracing Represented Thought in the French Novel (1800–1929)”, which was previously mentioned briefly and parenthetically, but deserved a more thorough engagement, as it helpfully lays out a number of considerations that are also central to our own point of departure with regard to the overall topic and the methods employed. Among these is the mapping of what Conroy terms “mental verbs” within literary texts of the period in question. In Conroy’s article, this quantitative approach is complemented by a detailed qualitative engagement with the development of French literature at the time, which would generally be an interesting way forward, but would have gone beyond the scope of this short report at the present time, given that our own project worked with 10 European languages with rather different developments in their literary histories (with some overlaps).

- *" **Another potential threat to validity is the composition of the corpus. I believe that this collection was developed for general purposes and not for this specific study. I don't know anything about the novels except their rough publication date and the fact that they were prominent enough to be digitized and selected for these collections. Do they represent modernism? Could the early ones have been chosen because they are avant garde (and thus happen to have more modern features), or the later ones because they are in a more conservative prestige-literary tradition?”***
- ***"A final word describing what COST Action “Distant Reading for European Literary History” is (project?) would help clarify for those unfamiliar."***

We have added more information about ELTeC, which was developed as part of the COST Action Distant Reading for European Literary History, in the main text (both in the introduction and in the section entitled “Data”) and hope to have drawn more attention to the papers that deal in detailed ways with the methodological background, choices, and criteria made in the context of corpus-building of ELTeC (Burnard et al.; Schöch et al.).

- ***“To account for the concern that studying all verbs would skew the results in favor of one category of verb, one could report general trends for all verbs and then individual trends for each category, thus allowing for both a broad and fine-grained study of the phenomenon.”***
- ***“The criteria by which the language experts selected the verbs for analysis is important.”***
- ***“I would include any visualizations referenced in the paper. Or, if there is only space for Figure 1, it seems it can be referred to for all of your analysis in this section.”***

In the revised version, we expanded the section concerning methodology and aimed to clarify our approach further, attempting to answer the important questions raised in the report in the process. We address individual comments below. All analyses discussed in the paper were performed both for all verbs jointly and for each category of verb separately. As an example of this approach, Figure 1 gives an overview of the relative frequency of all inner-life verbs over time per language, while Figure 2 shows the development of verbs in the affect category alone (for the extended French corpus).

- ***“I don't know how the confidence regions in Figure 1 are defined, but they look like bootstrap CIs, which would be a good option to increase statistical reliability. The second-order curve fit may be too smooth, I would rather see this as the actual numbers in a line plot with per-decade confidence bars.”***

We chose this method because higher polynomials fit the data more closely, retaining more details than a simple linear regression (the main text has been updated to reflect this term). We see this in the down-and-up curve for Romanian and the down-then-flat curve for Hungarian. The raw data, as well as preliminary visualisations created at earlier points in the process, can be found on GitHub: https://github.com/COST-ELTeC/innerlife/

- *“ **Why are the results for Serbian, referenced in the footnote, not included if they show similar trends?”***

We only received data for the first round of experiments with the Serbian corpus, not for the revised method based on the six categories of verbs derived from the Tompkins et al.

- ***“For the description of the third visualization category, what test statistic is calculated?"***

We added a footnote to the effect that we compared the two frequency distributions for the periods 1840-1869 and 1890-1919 using the (non-parametric) Mann-Whitney U-test as implemented in the SciPy library. **Stylistic comments and suggestions:** We are grateful for the detailed comments and line edits and took almost all suggestions relating to increased clarity, clarification, or usage of specific terms on board.

# Reviewer report: Reviewer response for version 1

**DOI:** 10.21956/openreseurope.17586.r35112

**Published:** 2023-09-25

**Authors:** Andreas van Cranenburgh, Noa Visser Solissa

**Affiliations:** 1 University of Groningen, Groningen, Groningen, The Netherlands; 2 Assistant professor of Digital Humanities and Information Sciences, University of Groningen, Groningen, Netherlands Antilles

This paper investigates the hypothesis that modernist novels focus more on the inner life of characters. The hypothesis is operationalized by counting the frequencies of inner life verbs. Contrary to what would be expected, modernist novels do not show a significantly higher frequency of such verbs.

 The paper is generally well written. However, some details are missing or are unclear.

 For example, the section of the selection of verbs talks about "nearest neighbors", but it is not clear by what method that was operationalized (the GitHub repository shows that word embeddings were used, apparently). Furthermore, several references to "categories" are made on page 1, without introducing how they are derived from the Theory of Mind framework.

 It is also unclear whether in the end there are different inner life verbs for each language, or whether translations of the same words are used. If only 3 verbs are selected per category for each language, the risk could be that these 3 words are not representative for the category.

 It would have been ideal to use an already validated lexicon for the project. For example, the LIWC lexicon has been validated in independent studies (and has been translated in several languages). In the current paper, it is not possible to separate the question of whether the lexicon is appropriate from the result for the research question.

 Another aspect is the statistical methodology. It is not made explicit how statistical significance was tested. Also, the Methods section announces that a linear regression line will be plotted, but Figure 1 and 2 actually show a polynomial regression. Why was polynomial regression chosen rather than linear?

 A suggestion: in Table 1 it would be more useful to show the percentage (or relative frequency) of inner life verbs, rather than absolute counts. There is a surprisingly large variance in word counts in the corpus, making absolute counts difficult to compare.

 Finally, one possible explanation for the results is that modernism gives more attention to inner life not through explicit inner life verbs, but through devices such as free indirect discourse, in which the thoughts of characters are described with explicitly marking them as such.

Is the work clearly and accurately presented and does it cite the current literature?

Partly

If applicable, is the statistical analysis and its interpretation appropriate?

Yes

Are the conclusions drawn adequately supported by the results?

Yes

Are all the source data underlying the results available to ensure full reproducibility?

Yes

Is the study design appropriate and is the work technically sound?

Yes

Reviewer Expertise:

Computational linguistics, computational literary studies.

We confirm that we have read this submission and believe that we have an appropriate level of expertise to confirm that it is of an acceptable scientific standard, however we have significant reservations, as outlined above.

# Response

**Published:** 2024-03-05

**Authors:** Tamara Radak

**Affiliations:** English and American Studies, University of Vienna, Vienna, Vienna, Austria

**Author response: **We are grateful to the reviewers for the detailed and observant report. We appreciate the productive feedback and clearly outlined questions, suggestions, and recommendations and respond to individual points made in the report, outlining how they are addressed in the revised version of the paper below. In the revised version, we expanded the section concerning methodology and aimed to clarify our approach further, attempting to answer the important questions raised in the report in the process. We address individual comments below.

- ***“For example, the section of the selection of verbs talks about "nearest neighbors", but it is not clear by what method that was operationalized (the GitHub repository shows that word embeddings were used, apparently). Furthermore, several references to "categories" are made on page 1, without introducing how they are derived from the Theory of Mind framework. It is also unclear whether in the end there are different inner life verbs for each language, or whether translations of the same words are used. If only 3 verbs are selected per category for each language, the risk could be that these 3 words are not representative for the category.”***

**Author response: **In the updated version, we have clarified that the approach using word embeddings was eventually abandoned, at least for the time being. We expanded on the section relating to the selection of verbs and the resulting lists, which are not identical, but comparable, while additionally also accounting for idiosyncracies between the different languages and corpora examined and ensuring that the verbs chosen have a high frequency within the respective corpus.

- ***“It would have been ideal to use an already validated lexicon for the project. For example, the LIWC.”***

**Author response: **We did not use the LIWC lexicon because it did not seem entirely appropriate for our setup, because 1) our aim was to arrive at a functionally equivalent set of words that best represent the individual corpora, not identical lists for each language based on a list of English terms that is then translated into other languages 2) we wanted to use the most frequent mental verbs in the individual corpora from the chosen categories, not mental verbs that might be the most common outside of the corpora, so we had a strict frequency criterion.

 In addition, we were doubtful as to whether LWIC has been validated for work with literary texts in earlier historical periods, such as the 19th century.

- ***“Another aspect is the statistical methodology. It is not made explicit how statistical significance was tested. Also, the Methods section announces that a linear regression line will be plotted, but Figure 1 and 2 actually show a polynomial regression. Why was polynomial regression chosen rather than linear?***

**Author response: **Regarding the issue of statistical significance, we added a footnote to the effect that we compared the two frequency distributions for the periods 1840-1869 and 1890-1919 using the (non-parametric) Mann-Whitney U-test as implemented in the SciPy library. We chose this method because higher polynomials fit the data more closely, retaining more details than a simple linear regression (the main text has been updated to reflect this term). We see this in the down-and-up curve for Romanian and the down-then-flat curve for Hungarian.

- ***"A suggestion: in Table 1 it would be more useful to show the percentage (or relative frequency) of inner life verbs, rather than absolute counts. There is a surprisingly large variance in word counts in the corpus, making absolute counts difficult to compare."***

**Author response: **We agree that this additional information would be helpful and have updated the table accordingly.

- ***“Finally, one possible explanation for the results is that modernism gives more attention to inner life not through explicit inner life verbs, but through devices such as free indirect discourse, in which the thoughts of characters are described with explicitly marking them as such.”***

**Author response: **In the revised version, we discuss this previously side-lined issue in more detail, both in the introductory part and the discussion of the results. 

 We added a paragraph discussing this issue in the Discussion section.

# Reviewer report: Reviewer response for version 1

**DOI:** 10.21956/openreseurope.17586.r34680

**Published:** 2023-09-19

**Authors:** Mark Algee Hewitt

**Affiliations:** 1 Department of English, Stanford University, Stanford, California, USA

This article seeks to use the European Literary Text Collection corpus (ELTeC) to put pressure on the widely held belief in Literary Studies that Modernist writing (writ large) directed attention to the inner lives of characters. Across the languages contained in the ELTeC, the authors track the usage of verbs that express characters’ interiority as a proxy for the attention to the inner life of characters that the authors hypothesize should increase as a function of time through the Modernist period. The results of the study, however, indicate that, barring some unique contexts, these verbs do not increase in a systematic way that would suggest that their increasing use is a hallmark of modernism.

 Overall, this research is compelling and aims to address a longstanding, yet largely unfounded, belief in Literary studies regarding the importance of interiority to 20 ^th^ century literary fiction. This is not only an interesting subject of study in and of itself, but it also provides a fascinating test case for the ELTeC corpus. I believe that the article has a great deal of merit and will make an important addition to our understanding of both the limits of computational literary study and the role of characterization in Modernism; however, there are a few, relatively minor, aspects of the study that I would like the authors to address before I can recommend the text for publication.

 Overall, my largest concern is the justification of the topic itself. I would not advocate for any substantive change to the methods employed by the authors, or their interpretations of their results; however, it will be important to include a bit more material justifying aspects of the study at the outset. For example, the authors suggest in their paragraph “Measuring modernism through ‘inner life’” that Modernism is not a universal phenomenon and does not take place across all languages at the same time. They do suggest that there are observable “processes implying formal innovation in national literatures throughout Europe at different times”, suggesting that they have underlying data which indicates the advent of Modernism in each language/cultural tradition represented by their corpus, but they do not make this data apparent. It is unclear if all of the languages represented in the corpus experience a “Modernist” period, or just some, and what kinds of formal innovation take place (these may be confounding variables to the study). It would be helpful for the authors to add in a little extra material explaining the periodization of the corpus they are using and the results that lead them to hypothesize the presence of “Modernist” literature in each of the languages that their corpus contains. This would allow for a more fine-grained hypothesis and help justify the goals of the study overall.

 Similarly, while the authors do an admirable job of explaining how they selected the verbs that may represent interiority, they do not explain how they treated these terms in the context of the different languages that the corpus contains. How were each of the verbs in the categories selected across languages? What efforts were made to ensure that the same selection of words would remain consistent despite the differences in meaning and frequency across the languages? Since part of the paper’s goal is to experiment with a multi-lingual corpus, this would be a very helpful detail to include. I’m also curious about how the authors counted the verbs. From the paper, it appears that, once the verbs were selected, the authors counted their incidence across the texts. While this is an understandable method, I would like some small explanation of why the frequencies of the verbs themselves were counted across the text as a whole, rather than being first filtered by their attachment to descriptions of character (through, for example, a dependency parse). Such a filter would ensure that the verbs are only being counted when they are attached to a named entity (presumably a character) rather than used in other contexts. Again, I’m not asking the authors to do this, simply to reference their choice not to.

 Aside from these two more substantive concerns, there are a small number of very minor points that the authors could augment or correct:

1. The Authors indicate that they use a “nearest neighbor” approach to building out their wordlist in one of the initial trials. Some more detail on what this approach was (a knn test, or something else) would be helpful.  
2. In figure 1, the authors visualize the trends in their results using a second order polynomial as a best fit line: why did authors choose this particular regression rather than a simple linear one?
3. In their description of Heuser and Le-Khac’s 2012 paper, the authors suggest that they found a trend to avoid telling in favor of showing from 1750-1950. The paper that they cite, however, only tracks this phenomenon in nineteenth century texts and their corpus stops in 1900. This is a relatively minor point, but considering that this paper is interested in modernism (which is often understood as a 20 ^th^ century phenomenon, this is an important caveat).

With these minor changes made (most of which just involves supplying the reader with some more information about the choices made by the authors), I would be happy to recommend this paper for indexing.

Is the work clearly and accurately presented and does it cite the current literature?

Yes

If applicable, is the statistical analysis and its interpretation appropriate?

Yes

Are the conclusions drawn adequately supported by the results?

Yes

Are all the source data underlying the results available to ensure full reproducibility?

Yes

Is the study design appropriate and is the work technically sound?

Partly

Reviewer Expertise:

I am an Associate Professor of Digital Humanities in a Literary Studies department (English) with over 20 years of experience in the field referenced by the article. My research focuses on quantitative and computational literary analysis, nineteenth-century literature and aesthetics, and the history of the novel.

I confirm that I have read this submission and believe that I have an appropriate level of expertise to confirm that it is of an acceptable scientific standard, however I have significant reservations, as outlined above.

# Response

**Published:** 2024-03-05

**Authors:** Tamara Radak

**Affiliations:** English and American Studies, University of Vienna, Vienna, Vienna, Austria

**Author response:** We are grateful to the reviewer for the thorough and perceptive report. We appreciate the productive feedback and clearly outlined questions, suggestions, and recommendations and respond to individual points made in the report, outlining how they are addressed in the revised version of the paper below. **Comments regarding overall topic, operationalization, periodization** • *“They do suggest that there are observable “processes implying formal innovation in national literatures throughout Europe at different times”, suggesting that they have underlying data which indicates the advent of Modernism in each language/cultural tradition represented by their corpus, but they do not make this data apparent. It is unclear if all of the languages represented in the corpus experience a “Modernist” period, or just some, and what kinds of formal innovation take place (these may be confounding variables to the study)”* In the revised version, we expanded on the connection between our project and Melanie Conroy’s 2014 paper “Before the ‘Inward Turn’: Tracing Represented Thought in the French Novel (1800–1929)”, which was previously mentioned briefly and parenthetically, but deserved a more thorough engagement, as it helpfully lays out a number of considerations that are also central to our own point of departure with regard to the overall topic and the methods employed. We have made more explicit and expanded on the concept of the “inward turn”, which is commonly posited as a hallmark feature of literary modernism and which was previously referenced in more implicit ways within our paper. • *“It would be helpful for the authors to add in a little extra material explaining the periodization of the corpus they are using and the results that lead them to hypothesize the presence of “Modernist” literature in each of the languages that their corpus contains.”* In the updated version, we provided examples of commonly accepted periodisations of literary modernism within some of the corpora, clarifying that the Portuguese corpus, which does not contain texts of writers who would unequivocally be termed “modernist” due to copyright issues, was included as a counter example and thus, a test case for the hypothesis. These periodisations are not based on data gathered via computational methods from literary corpora, but rather constitute a common consensus across literary histories of Europe (e. g. Walter Cohen’s 2017 book A History of European Literature: The West and the World from Antiquity to the Present). We acknowledge that questions of periodisation are not clear-cut or and that they represent a range of (often invisible) biases, preferences, and choices of their time (for example, with regard to the issue of canonisation). Both our own data (which are presented as a first step in a complex longer debate) and Conroy’s findings, along with other recent publications along similar directions suggest that computational methods and distant reading may be important tools for revisiting and critically questioning established narratives of literary history from fresh perspectives. **Comments relating to methodology** In the revised version, we expanded the section concerning methodology and aimed to clarify our approach further, attempting to answer the important questions raised in the report in the process.

- ***“I would like some small explanation of why the frequencies of the verbs themselves were counted across the text as a whole, rather than being first filtered by their attachment to descriptions of character (through, for example, a dependency parse). Such a filter would ensure that the verbs are only being counted when they are attached to a named entity (presumably a character) rather than used in other contexts. Again, I’m not asking the authors to do this, simply to reference their choice not to.”***

**Author response: **While this method would certainly have been helpful, it could not be done reliably across languages, at least at the present time. In future iterations of this and/or similar projects, using tools and such as those mentioned in Piper (2023) – bookNLP, super-sense tagging – could certainly be a productive way forward and potentially yield more fine-grained results. At present, we worked with existing tags and metadata that were developed in the context of ELTeC and the COST Action Distant Reading for European Literary History. **Other comments**

- ***“The Authors indicate that they use a “nearest neighbor” approach to building out their wordlist in one of the initial trials. Some more detail on what this approach was (a knn test, or something else) would be helpful.”  ***

**Author response: **Originally, we planned to compare two different methods – one based on frequencies of inner-life verbs and one based on seed words enriched through word embeddings. We clarified further that the second approach, involving word embeddings, was eventually abandoned because it was not possible to reliably filter out the ‘noise’, issues of polysemy, and similar issues. The raw data of this approach is, however, included in the extended data.

- ***“In figure 1, the authors visualize the trends in their results using a second order polynomial as a best fit line: why did authors choose this particular regression rather than a simple linear one.”***

**Author response: **We chose this method because higher polynomials fit the data more closely, retaining more details than a simple linear regression (the main text has been updated to reflect this term). We see this in the down-and-up curve for Romanian and the down-then-flat curve for Hungarian.

- ***“In their description of Heuser and Le-Khac’s 2012 paper, the authors suggest that they found a trend to avoid telling in favor of showing from 1750-1950. The paper that they cite, however, only tracks this phenomenon in nineteenth century texts and their corpus stops in 1900. This is a relatively minor point, but considering that this paper is interested in modernism (which is often understood as a 20th century phenomenon, this is an important caveat).”***

**Author response: **The original argument mentioned both Underwood’s and Heuser-Le Khac’s works, but the former was unintentionally omitted; the reference to Underwood’s 2019 publication *Distant Horizons*, which does cover this time period, is now reinstated.
